# Conscious and Resilient? Associations Between Temperament, Emotional Awareness, and Emotion Regulation Strategies in Youth

**DOI:** 10.5334/pb.1399

**Published:** 2026-02-27

**Authors:** Sarah Struyf, Ernst H. W. Koster, Marie-Lotte Van Beveren, Caroline Braet

**Affiliations:** 1 Department of Experimental Clinical and Health Psychology, Ghent University, Henri Dunantlaan 2, Ghent, Belgium; 2 Department of Developmental, Personality, and Social Psychology, Ghent University, Henri Dunantlaan 2, Ghent, Belgium

**Keywords:** Temperament, Emotional Awareness, Emotion Regulation, Youth

## Abstract

**Introduction:**

Emotion regulation (ER) plays a crucial role in children and adolescents’ emotional well-being. However, the use of adaptive strategies often remains challenging during early adolescence, partly because cognitive functions that support adaptive ER, such as executive functions, are still developing. In addition, individual differences, such as temperament (positive and negative emotionality) and emotional awareness, play a key role in shaping how youth engage with ER strategies. This study investigated how temperament and emotional awareness are associated with the use of adaptive and maladaptive ER strategies in youth, and whether emotional awareness moderates these relationships.

**Methods:**

In a cross-sectional design, 220 children and adolescents (age 8–15) from the general population completed self-report questionnaires measuring temperament, emotional awareness, and ER strategy use.

**Results:**

Analyses revealed a significant interaction between negative emotionality and emotional awareness in predicting maladaptive ER strategies. Contrary to expectations, emotional awareness did not act as a consistent protective factor in youth: those high in both negative emotionality and emotional awareness still reported greater use of maladaptive strategies.

**Conclusions:**

These findings suggest that emotional awareness plays a complex role in ER among youth with temperamental vulnerability. Rather than functioning as a uniformly protective factor, its influence appears to depend on emotional context and temperament.

## Introduction

Emotion regulation (ER) is essential for emotional well-being and refers to the processes by which individuals try to influence which emotions they experience, when and how they experience and express them (Gross, 1998). ER is widely recognized as a multifaceted skill including the use of ER strategies or the specific ways in which individuals actively reduce, maintain, or increase their emotional responses (Berking, 2017). Furthermore, ER is considered a transdiagnostic mechanism that plays a key role not only in promoting emotional well-being but also in the development and maintenance of various forms of psychopathology (Aldao et al., 2010). Importantly, ER strategies can broadly be divided into two categories based on their long-term effects. Typically, adaptive strategies such as reappraisal and problem solving are associated with emotional well-being long-term, whereas maladaptive strategies (e.g., rumination) are related to maladjustment and psychopathology in the long term (Aldao et al., 2010).

In the present study, ‘youth’ refers to children and adolescents aged 8–15, spanning late childhood through early adolescence, a period marked by significant changes in ER capacities. Since negative emotions are experienced more intensely during early adolescence, and key cognitive systems that support ER, including executive functions such as working memory, inhibition, and cognitive flexibility, are still developing, the effective use of adaptive strategies can be particularly challenging at this age (Somerville et al., 2010; Cracco et al., 2017). According to the maladaptive shift model (Cracco et al., 2017), early adolescence is marked by a dysfunctional shift, characterized by an increase in maladaptive strategies and a decline in adaptive strategies, driven by heightened emotional reactivity (Somerville et al., 2010). While adult psychopathology is often associated with the excessive use of maladaptive strategies (Aldao et al., 2010), findings in youth suggest that mental health problems are characterized by a dual pattern of elevated maladaptive strategy use and reduced adaptive strategy use (Braet et al., 2014; Schäfer et al., 2017). This highlights that, in late childhood and early adolescence, vulnerability to psychopathology reflects not only the presence of maladaptive responses but also a lack of effective adaptive regulation. Examining both adaptive and maladaptive strategies during this developmental period may therefore provide important insights into how emotional resilience can be supported.

However, children and adolescents vary considerably in their use of ER strategies, influenced by a range of individual factors. Among these, *temperament* and *emotional awareness* have each been identified as key contributors to individual differences in ER (Eastabrook et al., 2014; Van Beveren et al., 2020). Yet, to the best of our knowledge, these factors have mostly been examined in isolation, and little is known about how they interact. Investigating their combined influence may offer deeper insight into the mechanisms that support emotional resilience in youth. To explore these mechanisms further, we will first focus on temperament.

### Temperament and emotion regulation strategies

Individuals differ in how they experience and express emotions. According to Rothbart and Posner (2006), temperament refers to biologically based differences in how individuals respond to and interact with their environment. Although temperament is a broad construct encompassing multiple dimensions, the current study focuses specifically on *temperamental reactivity*. Rothbart and Bates (2006) distinguish two core components of temperamental reactivity: *Positive Emotionality* (PE) and *Negative Emotionality* (NE). These dimensions reflect stable tendencies in emotional responding that are observable from early childhood and remain relatively consistent over time. PE refers to the tendency to experience positive emotions such as joy, interest, and enthusiasm (Rothbart et al., 2000). It has been linked to the behavioral activation system (BAS; Gray, 1970) and is associated with the personality trait of extraversion later in life (Eysenck, 1967). In contrast, NE reflects a predisposition toward negative emotions, including anxiety, sadness, and anger (Rothbart et al., 2000), and has been related to the behavioral inhibition system (BIS; Gray, 1970) and neuroticism (Eysenck, 1967). Temperament is thought to provide a stable foundation for emotional development, shaping both the emergence of personality traits and ER processes (Rothbart et al., 2000). Moreover, temperament plays a key role in the development of psychopathology, with high NE identified as a general risk factor and low PE as a specific vulnerability marker for depression (Lonigan et al., 2003).

Given that PE and NE shape emotional experiences and reactivity, temperament has gained prominence as a framework for understanding individual differences in the development of ER strategy use (Rothbart & Sheese, 2007). From a theoretical perspective, children and adolescents are thought to regulate their emotions in ways that align with their temperamentally driven emotional sensitivities and tolerances (Thompson, 2007). Integrated cognitive-affective models provide a useful lens for understanding how PE and NE influence ER. For instance, the ABC model (Affective, Biological, Cognitive; Hyde et al., 2008), developed to explain gender differences in depression, links high NE to the use of maladaptive strategies such as rumination. In contrast, the broaden-and-build theory (Fredrickson, 2001; Tugade & Fredrickson, 2007) posits that PE broadens attention and thinking, thereby facilitating the development of adaptive strategies.

When linking these models to empirical research, numerous studies have reported associations between temperament and ER strategy use. High levels of NE have consistently been linked to greater use of maladaptive strategies overall (e.g., Van Beveren et al., 2016), and more specifically to rumination in youth (Van Beveren et al., 2019; Mezulis et al., 2011).

In addition, NE has been associated with reduced use of adaptive strategies (Van Beveren et al., 2018), whereas PE has been linked to more frequent use of adaptive strategies and lower engagement in maladaptive strategies across adolescence (Verstraeten et al., 2012) and young adulthood (Gross & John, 2003). Conversely, lower levels of PE are related to less frequent use of adaptive strategies (Gross & John, 2003; Harding et al., 2014; Van Beveren et al., 2018). In summary, youth with high NE and low PE show vulnerability in their use of ER strategies, with reduced use of adaptive strategies and, in the case of high NE, greater reliance on maladaptive strategies. In contrast, high PE is associated with more frequent use of adaptive strategies and reduced use of maladaptive strategies (Gross & John, 2003; Verstraeten et al., 2012; Van Beveren et al., 2018).

These patterns can be understood within a cognitive-affective framework that links emotional reactivity to the conditions under which ER unfolds. According to the broaden-and-build theory (Fredrickson, 2001), positive emotions broaden cognition and facilitate access to the resources needed for adaptive regulation. In line with this view, PE may support flexible, goal-directed ER by enhancing attentional control and working memory. Empirical findings partly support this account, with children showing better executive-function performance when experiencing higher levels of positive affect (Lo et al., 2025), although such effects are not consistently observed (e.g., Neubauer et al., 2019; Wang et al., 2021). Conversely, elevated NE may interfere with executive functions such as inhibition and working memory (Joormann & Gotlib, 2010), thereby increasing the likelihood that regulation unfolds in a more automatic, maladaptive manner (Van Beveren et al., 2018). Together, these accounts suggest that vulnerability in ER is not solely rooted in emotional reactivity itself, but also in individuals’ capacity to move beyond automatic responses and engage more deliberate forms of regulation.

Building on this distinction between automatic and more deliberate forms of ER, it has been suggested that additional factors may influence whether maladaptive regulatory tendencies are enacted or overridden. One such factor is *emotional awareness*, which has been proposed to facilitate the interruption of automatic patterns of maladaptive ER and to support the engagement of more deliberate regulatory responses (Berking, 2017). Notably, Tugade and Fredrickson (2007) suggest that even youth with temperamental vulnerabilities (i.e., high NE or low PE) retain the capacity to develop adaptive strategies, although this may occur less readily than in their more resilient peers. In this context, emotional awareness may represent an important factor that enables youth to move beyond automatic emotional responses and engage in more adaptive regulation.

### Emotional awareness and emotion regulation strategies

Emotional awareness refers to the ability to consciously recognize, label, and attend to one’s emotions in a nonjudgmental manner (Lane & Schwartz, 1987). According to the Adaptive Coping with Emotions (ACE) model (Berking, 2017), emotional awareness constitutes a crucial first step in the ER process and is essential for the effective implementation of adaptive ER strategies. It is proposed to support regulation in two key ways: first, by interrupting automatic tendencies to interpret emotional situations negatively, and second, by enabling individuals to evaluate their emotional experience and consider appropriate strategies for downregulation (Berking, 2017). In children and adolescents, heightened emotional awareness may help inhibit maladaptive responses when negative emotions are intense, thereby facilitating the deliberate use of adaptive strategies. Conversely, low emotional awareness has been linked to difficulties in effectively using adaptive strategies (Eastabrook et al., 2014). Without sufficient awareness and understanding of their emotional states, youth are more likely to rely on reactive processes, which may result in maladaptive regulation and increased vulnerability to psychopathology (Eastabrook et al., 2014; Van Beveren et al., 2019).

The proposed link between emotional awareness and ER has been examined in adults and, to a lesser extent, in youth. Eastabrook et al. (2014), for instance, investigated ER strategies in relation to emotional awareness and internalizing symptoms among adolescent girls (ages 13–16). They found that higher self-reported emotional awareness was positively associated with reappraisal and negatively associated with suppression. Similarly, Van Beveren et al. (2019) reported a positive association between emotional awareness and the use of adaptive strategies in youth (ages 8–15), although no significant association was found with maladaptive strategies. In contrast, De Witte et al. (2016) reported that higher interoceptive awareness, measured via heartbeat detection, was negatively associated with rumination and self-devaluation in youth (ages 9–16), but unrelated to adaptive strategy use. Although heartbeat detection tasks assess bodily rather than emotional awareness, interoceptive awareness may support awareness of internal emotional states.

In sum, two of the three studies (Eastabrook et al., 2014; Van Beveren et al., 2019) provide support for the notion that emotional awareness is an important skill for the use of adaptive ER strategies (Berking, 2017). In addition, two studies (Eastabrook et al., 2014; De Witte et al., 2016) suggest a negative association between emotional awareness, or closely related constructs such as interoceptive awareness, and maladaptive strategies. However, findings in youth remain limited and somewhat inconsistent. Moreover, none of these studies examined the role of temperament. Nevertheless, temperament, emotional awareness, and ER are likely to be interrelated.

### Temperament, emotional awareness, and emotion regulation strategies

Consistent with the distinction introduced above between more automatic and more deliberate forms of ER, maladaptive strategies are generally considered more reactive in nature and more strongly rooted in biologically based dispositions such as temperament (Fredrickson, 2001; Hyde et al., 2008), whereas the use of adaptive strategies typically requires more deliberate and conscious engagement (Berking, 2017; Van Beveren et al., 2019). Individuals with higher levels of emotional awareness are generally better able to tolerate and process intense emotional experiences (McRae et al., 2008). In this context, emotional awareness may function as a compensatory factor for youth with temperamental vulnerabilities, supporting more effective regulation in emotionally demanding situations. Examining the potential moderating role of emotional awareness may therefore provide valuable insight into individual differences in ER and help elucidate why some youth are able to regulate emotions adaptively despite underlying biological risk factors.

A small number of lab studies have examined the moderating role of emotional awareness in the relationship between negative emotional states and ER in adults. For instance, Füstös et al. (2012) demonstrated that individuals with higher emotional awareness were better able to regulate arousal associated with negative affect through adaptive strategies such as reappraisal. Similarly, Pollatos et al. (2015) found that higher emotional awareness enhanced the regulation of negative emotions in women, potentially by preserving limited cognitive resources involved in ER. In a related study, Szczygieł et al. (2012) showed that high emotional awareness buffered against errors during emotional information-processing tasks, thereby reducing the disruptive effects of negative emotions on cognitive-emotional functioning. Notably, when participants were explicitly instructed to use reappraisal, performance remained stable regardless of emotional awareness levels, suggesting that emotional awareness may be particularly relevant in more automatic or unstructured emotional contexts. Taken together, these findings suggest that emotional awareness can moderate the impact of emotional reactivity on regulation processes. However, further research is needed to clarify the mechanisms underlying this effect and to delineate the conditions under which emotional awareness plays a facilitating role.

In conclusion, research on emotional awareness in both adults and youth provides valuable insights into factors that support ER. However, its role in ER strategy use has rarely been examined in relation to temperamental differences, and existing findings remain limited and somewhat inconsistent. Moreover, empirical studies simultaneously considering the influence of both PE and NE on adaptive and maladaptive ER strategies in youth are scarce. The current study addresses these gaps by examining how temperament and emotional awareness jointly relate to ER strategy use in youth, with the aim of identifying factors that may foster resilience in the context of emotional vulnerability.

### The current study

This study aimed to investigate how children’s and adolescents’ temperament and emotional awareness are associated with their use of ER strategies. Building on theoretical models in both adult (e.g., Berking, 2017; Tugade & Fredrickson, 2007) and youth populations (Hyde et al., 2008), as well as empirical findings in youth (e.g., Van Beveren et al., 2019, 2020), we examined both direct associations and interaction effects. Specifically, we expected higher levels of PE to be associated with more frequent use of adaptive strategies and less frequent use of maladaptive strategies, whereas higher levels of NE were hypothesized to relate to reduced use of adaptive strategies and increased use of maladaptive strategies. Emotional awareness was expected to be positively associated with adaptive strategy use and negatively associated with maladaptive strategy use. Importantly, we also tested whether emotional awareness moderated the associations between temperament and ER strategies. We hypothesized that higher emotional awareness would strengthen the positive association between PE and adaptive strategy use and strengthen the negative association between PE and maladaptive strategy use. Similarly, emotional awareness was expected to facilitate adaptive strategy use among youth with high NE and to buffer the association between NE and maladaptive strategy use. By examining these interaction effects, the present study aims to provide new insights into how temperament and emotional awareness jointly shape ER patterns during late childhood and the transition into early adolescence. Although the cross-sectional design precludes conclusions about developmental change, focusing on this age range offers important context for understanding how these associations manifest during a period marked by substantial emotional and cognitive development.

## Materials & Methods

### Participants

The sample consisted of 220 Dutch-speaking children and adolescents (53.3% girls), aged between 8 and 15 years (M_age_ = 11.92, SD = 1.80), all residing in Belgium. Socioeconomic status (SES), based on parents’ educational level and occupation (Hollingshead, 1975), was primarily middle (52.7%) or upper-middle class (37.3%), with a small proportion classified as lower-middle (5.5%) or lower class (0.5%). Regarding family composition, 78.6% of participants lived with both parents, 9.1% with one parent and their new partner, 5.5% with their mother only, 0.9% with their father only, and 5.5% alternated between both parents due to co-parenting arrangements.

The current study used data from the fourth phase (T4) of the *Generation 2020* project, a longitudinal research initiative focused on emotional well-being and school readiness in youth (see Van Beveren et al., 2019). The project included all children from 4th to 8th grade attending public schools in Deinze (Flanders, Belgium) and was approved by the Ethical Committee of the Faculty of Psychology and Educational Sciences at Ghent University. Although the broader project included multiple assessment waves, the present study is based exclusively on data collected during T4, resulting in a cross-sectional design. At this stage, a subset of 220 children and adolescents was visited at home by trained psychology students, who administered all questionnaires under standardized conditions. This number corresponded to the available pool of 220 psychology students, with each student assigned to one participating child or adolescent.

### Measures

#### Reactive Temperament

Positive Emotionality (PE) and Negative Emotionality (NE) were measured using the Positive and Negative Affectivity Schedule for Children (PANAS-C; Laurent et al., 1999; Dutch translation: De Bolle, 2007). The PANAS-C is a self-report instrument for children (age 7–14) containing 30 items assessing the participants’ general experience of emotion. With 15 items for the NE subscale and 15 items for the PE subscale, participants report to which extent they usually experience each emotion on a 5-point Likert scale. The PANAS-C has good psychometric qualities, including good convergent and discriminant validity (Laurent et al., 1999). Cronbach’s alphas were α = .88 and α = .58 for NE and PE respectively. While the internal consistency of the PE scale was lower than the conventional threshold, the scale was retained given its theoretical relevance, established use in prior studies, and the fact that α values can be sensitive to sample characteristics.

#### Emotional Awareness

Emotional awareness was assessed with the Awareness subscale of the Dutch version of the Difficulties in Emotion Regulation Scale (DERS; Gratz & Roemer, 2004; Neumann et al., 2010). The subscale consists of six self-report items that measure individuals’ attention to, and awareness of, their own emotional responses on a 5-point Likert scale ranging from 1 (almost never) to 5 (almost always). In the original scoring, this subscale is reverse-coded so that higher scores reflect less awareness. In the present study, however, we used the raw (non-reversed) scores, thereby treating this dimension as emotional awareness (i.e., higher scores indicate greater awareness), consistent with previous research in youth (e.g., Van Beveren et al., 2019). The Dutch DERS has demonstrated adequate psychometric properties in adolescents (Neumann et al., 2010), although the Awareness subscale is known to have weaker reliability compared to other subscales (Bardeen et al., 2012; Neumann et al., 2010). Cronbach’s alpha for the Awareness subscale in the present sample was .71.

#### Emotion Regulation Strategies

ER strategies were assessed using the FEEL-KJ questionnaire for emotion regulation in children and adolescents (Braet et al., 2013). The FEEL-KJ is a 90-item self-report questionnaire assessing the use of various adaptive, maladaptive, and external ER strategies in response to fear, sadness and anger. Participants aged 8 to 18 years can rate each item on a 5-point Likert scale from (1) ‘almost never’ to (5) ‘almost always’. In the current study, only the total adaptive and total maladaptive ER strategies subscales were considered. Total scores on these scales were calculated over all three emotions and represent general dispositions to adaptively or maladaptively cope with these emotions. The adaptive subscale comprises the strategies behavioral problem solving, cognitive problem solving, forgetting, acceptance, distraction, positive refocusing, and reappraisal. The maladaptive subscale includes the strategies giving up, aggression, rumination, self-devaluation, and withdrawal. The FEEL-KJ has proven to be a valid and reliable instrument (Braet et al., 2013; Cracco et al., 2015). Cronbach’s alphas for the adaptive and maladaptive subscale were .95 and .86 respectively.

### Data analytic strategy

Prior to testing the study’s hypotheses, preliminary analyses were conducted to examine bivariate correlations among all variables and to assess potential effects of gender and age on ER strategy use. Given previous research identifying age and gender differences in children and adolescents’ use of ER strategies (Cracco et al., 2017; Hyde et al., 2008; Mezulis et al., 2011), these variables were considered as covariates in the main analyses when significantly related to the outcome variables. To test the hypotheses, multiple linear regression analyses were performed, with statistical significance set at α < .05. All variables were standardized prior to the interaction analyses. Four regression models were tested in total. Where applicable, age and/or gender were entered in Step 1, followed by the main effects of temperament (i.e., PE, NE) and emotional awareness in Step 2. In Step 3, the interaction term between temperament and emotional awareness was added. Separate models were tested for adaptive and maladaptive ER strategies. All analyses were conducted using IBM SPSS Statistics version 27.0. Due to minimal missing data, regression models were run with 219 participants (listwise deletion).

## Results

### Descriptives and preliminary analyses

Means, standard deviations, and correlations between all variables are presented in Table 1. Preliminary analyses showed that age was significantly associated with maladaptive ER strategies (*β* = .19, *t*(218) = 2.85, *p* < .01), indicating that the use of such strategies tends to increase with age during early adolescence. Gender, however, was not significantly related to maladaptive strategies (*β* = .12, *t*(218) = 1.78, *p* = .077). For adaptive strategies, neither age (*β* = .03, *t*(218) = .42, *p* = .674) nor gender (*β* = .10, *t*(218) = 1.53, *p* = .127) showed significant associations. Based on these findings, age was included as a control variable only in the analyses concerning maladaptive ER strategies. This approach ensured parsimony and avoided unnecessary adjustment in models where age was not significantly related to the outcome. Assumption checks indicated that study variables and regression residuals were approximately normally distributed, with only minor deviations at the tails. Scatterplots supported homoscedasticity, and Cook’s distance values were all well below 1.0, suggesting that outliers did not exert undue influence. No concerns regarding multicollinearity emerged, with tolerance values >.95, VIF values close to 1, and condition indices <30. Together, these results support the adequacy of the regression assumptions.

**Table 1:** Descriptive statistics and correlations between temperament, emotional awareness and emotion regulation strategies.


VARIABLES	1	2	3	4	*M* (SD)	MIN	MAX

**1. NE**					32.15 (9.10)	15.00	64.00

**2. PE**	–.08				45.44 (6.82)	22.00	95.00

**3. EA**	.06	.16*			17.99 (4.50)	6.00	28.00

**4. AD ER**	–.20**	.26**	.30**		135.60 (25.83)	78.00	210.00

**5. MAL ER**	.56**	–.09	.17*	–.02	71.91 (14.58)	31.00	117.00


*Note. NE* negative emotionality, *PE* positive emotionality, *EA* emotional awareness, *AD ER* total adaptive ER strategies, *MAL ER* total maladaptive ER strategies. * *p* < .05; ** *p* < .01.

### Positive Emotionality, Emotional Awareness, and Emotion Regulation Strategies

As shown in Table 2, regression analyses revealed a significant main effect of PE on adaptive ER strategy use, indicating that children and adolescents high in PE reported greater use of adaptive strategies. Consistent with expectations, emotional awareness (hereafter abbreviated as EA, abbreviation used throughout the Results section for clarity) also showed a significant positive association with adaptive strategies. However, no moderation effect emerged: the PE × EA interaction did not explain additional variance beyond the main effects.

**Table 2:** Summary of hierarchical regressions concerning research model one and two.


	N	B	SE B	*β*	T	F	R^2^ CHANGE

**DV: adaptive ER strategies (AD ER)**							

**STEP 1**							

PE	219	5.60	1.65	.22	3.38***		

EA	219	6.91	1.65	.27	4.19***	17.10***	.14***

**STEP 2**							

PE	219	5.36	1.68	.21	3.19**		

EA	219	6.92	1.65	.27	4.19***		

PE × EA	219	1.14	1.65	.04	.69	11.53***	.002

**DV: maladaptive ER strategies (MAL ER)**							

**STEP 1**							

Age	219	.11	.04	.19	2.85**	8.14**	.04**

**STEP 2**							

Age	219	.08	.04	.15	2.19*		

PE	219	–.08	.07	–.08	–1.10		

EA	219	.16	.07	.16	2.29*	4.66**	.03

**STEP 3**							

Age	219	.08	.04	.15	2.18*		

PE	219	–.07	.07	–.07	–.97		

EA	219	.16	.07	.16	2.28*		

PE × EA	219	–.04	.07	–.04	–.61	3.58**	.002


*Note. PE* positive emotionality, *EA* emotional awareness, *AD ER* total adaptive ER strategies, *MAL ER* total maladaptive ER strategies. * *p* < .05; ** *p* < .01; *** *p* < .001.

Regarding maladaptive strategies, no significant main effect was found for PE. Unexpectedly, EA was positively associated with maladaptive strategy use, suggesting that higher EA may coincide with greater use of maladaptive strategies. The interaction term (PE × EA) was again non-significant.

### Negative Emotionality, Emotional Awareness, and Emotion Regulation Strategies

Table 3 presents the regression models examining NE, EA, and adaptive ER strategies. As hypothesized, NE was negatively associated with adaptive strategy use indicating that children and adolescents high in NE reported using fewer adaptive strategies. EA also showed a significant positive association with adaptive strategies. However, no significant interaction was found: the NE × EA term did not predict additional variance.

**Table 3:** Summary of hierarchical regressions concerning research model three and four.


	N	B	SE B	*β*	T	F	R^2^ CHANGE

**DV: adaptive ER strategies (AD ER)**							

**STEP 1**							

NE	219	–.21	.06	–.21	–3.37***		

EA	219	.31	.06	.31	4.95***	17.04***	.14***

**STEP 2**							

NE	219	–.21	.06	–.21	–3.36***		

EA	219	.31	.07	.31	4.81***		

NE × EA	219	–.01	.07	–.01	–.09	11.31***	.000

**DV: maladaptive ER strategies (MAL ER)**							

**STEP 1**							

Age	219	.11	.04	.19	2.85**	8.14**	.04**

**STEP 2**							

Age	219	.08	.03	.14	2.48*		

NE	219	.54	.06	.54	9.86***		

EA	219	.12	.06	.12	2.08*	38.57***	.31***

**STEP 3**							

Age	219	.08	.03	.15	2.63**		

NE	219	.54	.06	.54	9.75***		

EA	219	.09	.06	.09	1.60		

NE × EA	219	–.12	.06	–.12	–2.07*	30.44***	.01*


*Note. NE* negative emotionality, *EA* emotional awareness, *AD ER* total adaptive ER strategies *MAL ER* total maladaptive ER strategies. * *p* < .05; ** *p* < .01; *** *p* < .001.

The regression models for maladaptive ER strategies also appear in Table 3. NE was significantly and positively associated with maladaptive strategy use as consistent with expectations. Additionally, EA showed a small but significant positive association with maladaptive strategies suggesting that higher EA may coincide with greater use of maladaptive strategies.

**Figure 1:**
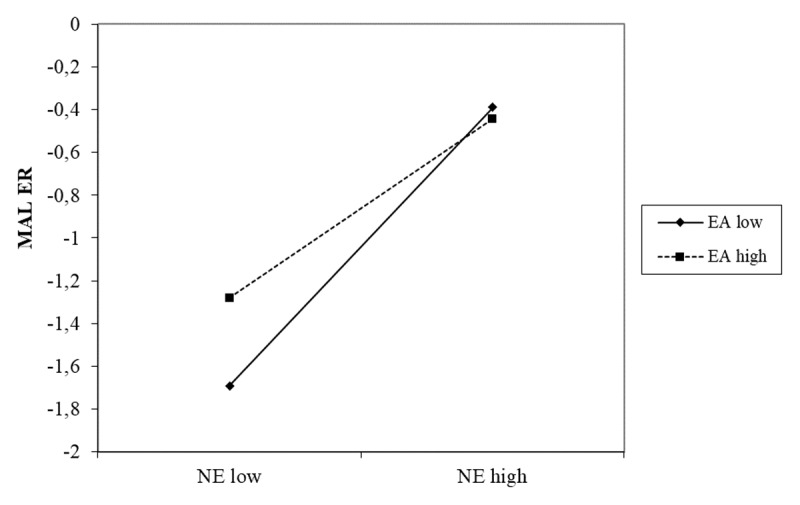
The interaction effect of negative emotionality (NE) and emotional awareness (EA) on maladaptive ER strategies (MAL ER).

In the final step, the NE × EA interaction significantly predicted maladaptive strategy use, explaining variance beyond the main effects. As visualized in Figure 1, simple slopes were plotted at high (+1 SD) and low (–1 SD) values of EA and NE. Maladaptive strategy use was lowest among youth with both low NE and low EA. However, as NE increased, maladaptive strategies rose sharply, particularly among those low in EA. At high levels of NE, maladaptive strategy use was elevated across groups, though slightly higher for those low in EA.

## Discussion

This study examined how temperament and emotional awareness relate to ER strategies in youth, and whether emotional awareness moderates these associations. The findings are discussed below in light of their theoretical implications, directions for future research, and the study’s strengths and limitations.

### Temperament and Emotion Regulation strategies

Overall, the findings align with previous research on the role of PE and NE in ER strategy use among youth (e.g., Van Beveren et al., 2016). Specifically, PE was positively associated with adaptive strategies, supporting its role as a resilience factor (Tugade & Fredrickson, 2007). Youth high in PE may be more attuned to positive information in daily life, which is thought to be linked to greater use of adaptive strategies such as reappraisal and positive refocusing. In contrast, PE was not significantly associated with reduced use of maladaptive strategies. This corresponds with earlier research, which has primarily focused on PE in relation to adaptive strategies. Nonetheless, some studies have reported associations between PE and lower use of specific maladaptive strategies, such as suppression (Gross & John, 2003) and dampening (Verstraeten et al., 2012). These response-focused strategies aim to suppress or down-regulate (positive) emotions and may not be fully captured by broader measures of maladaptive ER. Since the present study focused on regulation of negative emotions, future research could further clarify the potential role of PE in regulating positive emotions. Importantly, the internal consistency of the PE scale was relatively low in our sample (α = .58). This may have introduced additional variability in the scores, potentially weakening associations with ER strategies and partly explaining the absence of significant moderation effects involving PE. Nonetheless, the observed positive link between PE and adaptive strategy use is consistent with theoretical expectations and previous research.

Regarding NE, the results show that higher levels were associated with greater use of maladaptive strategies. This aligns with theoretical accounts linking elevated NE to maladaptive ER tendencies, potentially because heightened negative affect can disrupt attentional and cognitive processes that support deliberate regulation (e.g., Joormann & Gotlib, 2010). Children and adolescents high in NE tend to be more reactive to negative stimuli and may more frequently report the use of maladaptive strategies when dealing with intense emotional states (Mezulis et al., 2011). NE was also negatively associated with adaptive strategy use, consistent with earlier findings linking NE to a general lack of adaptive responses (Van Beveren et al., 2018). Given that effective adaptive regulation has been associated with cognitive flexibility and a broad attentional scope, elevated NE may hinder the use of such strategies (Fredrickson, 2001; Van Beveren et al., 2020). Consequently, youth with higher levels of NE tend to rely more on maladaptive strategies and engage less in adaptive ones, underscoring NE’s role as a vulnerability factor in ER.

### Emotional Awareness and Emotion Regulation Strategies

As previously noted, adaptive strategies may require deliberate activation of cognitive resources, whereas maladaptive strategies are thought to occur more automatically (Fredrickson, 2001; Hyde et al., 2008; Van Beveren et al., 2019). This distinction reflects two partially distinct regulatory systems: automatic, bottom-up forms of regulation that are more reactive to emotional cues, and effortful, top-down forms that require cognitive control and conscious awareness (Gross, 2015; Gyurak et al., 2011). Within this framework, we hypothesized that emotional awareness would play a key role in ER. The results partially supported this assumption.

First, emotional awareness was strongly and positively associated with adaptive strategy use, consistent with previous research (e.g., Van Beveren et al., 2019) and theoretical models of ER (Berking, 2017). Strategies such as cognitive reappraisal involve reframing emotional experiences and may depend on a clear awareness of emotional triggers and responses. Youth reporting higher levels of emotional awareness may therefore be more likely to engage in such cognitively demanding forms of regulation.

Second, contrary to our hypotheses, emotional awareness was also positively associated with maladaptive strategy use. Although this association was weaker than that with adaptive strategies, it suggests that youth who are more attentive to their emotions also tend to engage more in maladaptive responses. This finding contrasts with earlier studies reporting a negative link between emotional awareness and maladaptive strategies (e.g., De Witte et al., 2016; Eastabrook et al., 2014) but partially aligns with Ost et al. (2019). In that study, emotional awareness was measured using both the AQC (reflecting alexithymia, i.e., difficulties identifying and verbalizing emotions) and the DERS Awareness subscale (capturing attentional focus on emotions). Lower alexithymia was related to more adaptive and less maladaptive strategy use, whereas higher awareness was linked to greater use of both adaptive and maladaptive strategies. These discrepancies likely reflect conceptual differences between the constructs, corresponding to different stages in the ACE model (Berking, 2017): emotional awareness involves noticing and attending to emotions (step one), while alexithymia reflects difficulties identifying and labeling them (step two). As noted by Boden and Thompson (2017), emotional awareness is multidimensional, comprising distinct yet interacting facets: *attention to emotions* and *emotional clarity*. While attending to emotions can facilitate regulation, excessive attention without sufficient clarity may increase vulnerability to maladaptive responses such as rumination (see also Vine et al., 2014). Recent meta-analytic evidence supports this view: Kim et al. (2025) found that emotional clarity consistently predicts lower distress, whereas attention is adaptive only when paired with clarity. When clarity is low, high attention instead relates to greater distress, underscoring that the adaptiveness of emotional awareness depends on a balanced integration of its facets. In this sense, youth who are highly attentive but lack clarity may become overwhelmed by their emotions, reducing regulatory effectiveness.

Developmental factors may further account for this pattern. Emotional awareness and metacognitive insight typically increase during late childhood and early adolescence, while the ability to flexibly select context-appropriate strategies is still maturing (Crone & Dahl, 2012; Zimmermann & Iwanski, 2014). Youth who are more attuned to their emotions may therefore experiment with a broader range of strategies, both adaptive and maladaptive, as part of learning what works for them (Fombouchet et al., 2023). In this sense, greater emotional awareness may signal engagement in ER rather than regulatory success per se.

Taken together, these findings suggest that emotional awareness supports the early stages of ER but is not a standalone protective factor. Its adaptive potential likely depends on the integration of complementary skills such as emotional clarity, acceptance, and cognitive flexibility, and on developmental maturity in applying them effectively. In youth, higher emotional awareness may thus represent an emerging readiness to engage with emotions, marking a crucial but still evolving step toward regulatory competence.

### Temperament, Emotional Awareness, and Emotion Regulation Strategies

Building on the idea that emotional awareness supports the conscious use of adaptive strategies and that temperament shapes individual differences in ER tendencies, we expected these factors to be closely interrelated. Specifically, emotional awareness was hypothesized to act as a compensatory factor, enabling youth with temperamental vulnerability to regulate emotions more adaptively. However, the findings offered a more nuanced picture than anticipated.

No interaction was found between PE and emotional awareness: higher levels of both did not jointly predict greater adaptive strategy use or reduced maladaptive use. This may partly reflect that our analyses relied on composite scores of adaptive strategies, whereas cognitive strategies such as reappraisal or problem solving may be more sensitive to individual differences in PE and emotional awareness (Van Beveren et al., 2018, 2019). Additionally, interactions may emerge more clearly when NE is also considered, as youth high in NE and low in PE may be less equipped to access cognitively demanding regulation strategies (Van Beveren et al., 2018). Future research with larger samples could examine this in more detail.

In contrast, a significant interaction emerged between NE and emotional awareness in predicting maladaptive strategy use. Contrary to expectations, emotional awareness did not clearly buffer the effect of NE. Rather, maladaptive strategy use appeared primarily driven by NE: youth high in NE reported the highest maladaptive use overall. Differences between high and low emotional awareness were small in this group, suggesting that emotional awareness exerted, at most, a minor attenuating effect. When NE was low, however, higher emotional awareness was associated with somewhat greater maladaptive use compared to low awareness. This pattern indicates that while emotional awareness may slightly modulate the expression of NE, it does not fundamentally alter its impact on maladaptive ER. Overall, these findings suggest that emotional awareness is not uniformly protective but interacts with temperamental context in complex ways.

One possible explanation is that heightened emotional awareness without sufficient clarity may intensify emotional reactivity. Adolescents who are highly attuned to their emotions but struggle to interpret them may become overly focused on negative states, fostering rumination and difficulties disengaging from distress (Boden & Thompson, 2017; Koster et al., 2011; Vine et al., 2014). From the perspective of the disengagement hypothesis (Koster et al., 2011), such over-attention to internal states may hinder attentional shifts away from negative affect, thereby maintaining maladaptive regulation cycles. Thus, emotional awareness alone may not provide regulatory benefits and may even contribute to greater emotional entanglement when not accompanied by sufficient clarity or cognitive control.

Developmental factors may further shape this pattern. During late childhood and early adolescence, emotional awareness typically increases, while higher-order regulatory abilities are still developing (Crone & Dahl, 2012; Zimmermann & Iwanski, 2014). As a result, youth with heightened awareness may be motivated to regulate emotions but lack the cognitive ability or experience to do so effectively. This developmental imbalance, high awareness combined with immature regulation capacities, could partly explain why greater emotional awareness sometimes coincides with higher maladaptive strategy use. Another possibility is that adolescents with high emotional awareness are simply more attuned to their emotional life and regulation attempts, making them more likely to report a wider range of strategies, regardless of their actual frequency or effectiveness.

Taken together, these findings highlight that emotional awareness plays a nuanced role in youth ER. While it may slightly temper maladaptive responses under high emotional reactivity, it may also heighten focus on negative states when clarity or control are insufficient, underscoring that awareness alone is not enough for adaptive regulation.

## Theoretical and Clinical Implications

Contrary to initial expectations, the findings indicate that high emotional awareness is associated with both adaptive and maladaptive ER strategies. This suggests that emotional awareness alone may not be sufficient to foster exclusively adaptive regulation. Other ER skills, such as those outlined in the ACE model (Berking, 2017), likely also shape how emotions are regulated. Future research examining a broader set of ER competencies could offer further insight into individual differences in ER profiles. High NE consistently emerged as a vulnerability factor for maladaptive ER, regardless of emotional awareness levels. Further studies could explore which cognitive processes pose specific challenges for children and adolescents high in NE. In addition, exploring more complex interactions such as those involving PE, NE, and emotional awareness, may be valuable. Prior research (Van Beveren et al., 2018) suggests that youth with high NE and low PE may lack cognitive resources needed for adaptive regulation. Investigating whether emotional awareness can compensate for this profile could clarify whether these children and adolescents are able to engage in more constructive strategies.

Beyond theoretical relevance, the findings highlight the importance of differentiating between emotional competencies in clinical practice. While emotional awareness plays a role in ER strategy use, its impact may depend on temperament. Assessment tools like the DERS and FEEL-KJ can help identify ER strengths and difficulties, while temperament measures (e.g., PANAS-C) may reveal underlying vulnerabilities. Such an integrated approach can support more personalized interventions. For children and adolescents with elevated NE and high emotional awareness, increased awareness may intensify focus on negative states without necessarily improving regulation. In such cases, interventions may benefit from emphasizing skills beyond emotional awareness such as strategy selection, flexibility, or behavioral implementation (Berking, 2017). Integrating these elements into clinical practice may enhance the precision and effectiveness of ER training for youth.

## Limitations and Future Research

Several strengths of this study are worth highlighting. First, it focuses on individual differences in early-stage processes relevant to ER (e.g. emotional awareness) rather than solely on the strategies themselves, which are often conceptualized as the end point of regulation. This approach provides insight into earlier phases of ER functioning and related vulnerabilities or strengths (Berking, 2017). Second, the study targets a young population, contributing to a relatively underexplored area despite adolescence being a critical period for the development of ER skills (Hyde et al., 2008). Third, we focused on broad indices of adaptive and maladaptive ER strategies, in order to capture general patterns of regulatory tendencies. This approach complements prior work that has often emphasized isolated strategies such as rumination (Mezulis et al., 2011) by providing a more comprehensive perspective on ER in youth. Finally, the inclusion of PE adds value, as most previous work has emphasized the role of NE in maladaptive ER.

Despite these contributions, several limitations should be acknowledged. All constructs were assessed using self-report measures, which may be susceptible to social desirability, shared method variance, and recall bias (Paulhus, 2002), and may capture only part of the regulatory process given that ER can occur outside conscious awareness (Gyurak et al., 2011). Several measurement-related considerations are also relevant. Although an authorized Dutch translation of the PANAS-C was used (De Bolle, 2007), this version has not yet been formally validated in a Dutch child and adolescent sample. Moreover, the internal consistency of the PE subscale was relatively low (α = .58), which may have attenuated associations with ER strategies and limited the detection of interaction effects. Findings involving PE should therefore be interpreted cautiously. Emotional awareness was assessed using the Awareness subscale of the DERS, which has shown applicability in adolescent samples (Neumann et al., 2010) but has been criticized for weaker psychometric properties relative to other subscales (Bardeen et al., 2012). Conceptually, this subscale primarily captures attentional and acknowledgment aspects of emotional awareness rather than more elaborative facets such as emotional clarity or differentiation, and thus represents only part of the broader construct. Finally, the cross-sectional design precludes conclusions about directionality, and the use of composite scores for adaptive and maladaptive strategies may obscure strategy-specific effects. Future research would benefit from longitudinal, fine-grained, and multi-method approaches, including behavioral tasks, psychophysiological measures, or ecological momentary assessment, to capture emotional processes more comprehensively.

## Data Accessibility Statement

The data that support the findings of this study are available on request from the corresponding author.

## Author Information

***Sarah Struyf***, Master’s degree in Psychology. PhD student and teaching assistant at Ghent University at the Psychopathology and Affective Neuroscience Lab (PANlab).

***Prof. Dr. Ernst Koster***, PhD in Psychology. Full professor at Ghent University and PI at the Psychopathology and Affective Neuroscience Lab (PANlab). He has published research in the area of clinical psychology, experimental psychopathology, depression and rumination.

***Dr. Marie-Lotte Van Beveren***, PhD in Psychology. Clinical psychologist at AZ Sint-Lucas Ghent and post-doctoral researcher at Ghent University. She has authored publications in the area of clinical developmental psychology, depression, temperament and emotion regulation.

***Prof. Dr. Caroline Braet***, PhD in Psychology. Professor Emerita at Ghent University. She was the PI at the Clinical Child and Adolescent Psychology (CCAP) Lab and remains affiliated with the lab, contributing to ongoing research and mentorship. She has published research in the area of clinical developmental psychology, depression, and emotion regulation in youth.
